# Using the ATra Black Box to Improve Public Health Data Linkages and Analytics in the DC Cohort Longitudinal HIV Study: Viewpoint on the Process and Findings

**DOI:** 10.2196/77119

**Published:** 2025-08-14

**Authors:** Anne Giuranna Rhodes, Maria Jaurretche, Lisa Mele, Daniel Jarris, Paige Kulie, Shannon Barth, Miranda Smith, J Smart, Amanda D Castel, DC Cohort Executive Committee

**Affiliations:** 1 Office of the Senior Vice President for Research, Georgetown University, 2115 Wisconsin Ave, Suite 6055, Washington, DC, 20057, United States, 1 8043356698; 2 District of Columbia Department of Health, Washington, DC, United States; 3 Biostatistics Center, George Washington University, Washington, DC, United States; 4 Department of Epidemiology, The Milken Institute School of Public Health, George Washington University, Washington, DC, United States

**Keywords:** public health, data sharing, surveillance, HIV, deduplication, linkage

## Abstract

The DC Cohort is a longitudinal HIV cohort study of people with HIV receiving care at 14 clinical sites in Washington, DC, led by George Washington University. Data are routinely linked to the DC Department of Health (DC Health) HIV surveillance databases to increase data completeness and accuracy and to help identify people with HIV enrolled at multiple sites. The ATra Black Box (Black Box henceforth) is a novel privacy technology developed by Georgetown University, which is currently deployed in 40 public health jurisdictions. The Black Box provides a secure mechanism to link private health information across data systems. The Black Box was modified for the purposes of linking data from the DC Cohort to DC Health surveillance data and increasing the ease, feasibility, accuracy, and timeliness of future linkages. These modifications included providing deidentified data to George Washington University and developing analytic code to compare data between the DC Cohort and DC Health to report on data discrepancies. This paper reports on the results of the initial linkage using the Black Box. DC Cohort data on all consented participants from January 2011 through September 2022 were submitted to the Black Box. Simultaneously, all DC Health HIV surveillance data were also submitted to the Black Box. The data were matched using a predetermined algorithm, match-level scores were assigned, and matches were manually verified. The new Black Box graphical user interface allows users to check files for errors and easily track the Black Box processes and provides analytic plugins for running SAS code. A total of 9744 records of DC Cohort participants were submitted for matching to DC Health. Of these, 9060 participants (93.0%) matched to surveillance data and were validated through manual review. Match-level scores ranged from 20 to 100, and the validation found that scores of 61 and above were true matches. The SAS output files provided information on missing or conflicting data, including lab records, date of HIV diagnosis, and other key demographics. The linkage resulted in the addition of 48,970 CD4 T-lymphocyte counts, 33,413 viral load lab records, and 767 previously unrecognized deaths. Among the DC Cohort participants, 470 were enrolled at more than one site and 17 at more than two sites. The implementation of the Black Box for sharing DC Cohort and DC Health data resulted in better capture of HIV lab records, improved vital status information, and enhanced characterization of care patterns for people with HIV enrolled in the DC Cohort. Future linkages will include DC Health data on diagnoses of sexually transmitted infections, hepatitis, and tuberculosis.

## Introduction

The “Ending the HIV Epidemic in the US” initiative, introduced by the US Department of Health and Human Services in 2019, and the National HIV Strategic Plan: 2021‐2025, introduced by the US Department of Health and Human Services in 2021, focus on reducing new HIV infections and ensuring people with HIV are in care and achieve viral suppression [1,2]. They track progress through metrics, such as linkage and retention in HIV care and viral load suppression, using a variety of sources, including health department surveillance data and electronic health records (EHRs).

The DC Cohort is a longitudinal, prospective, clinic-based cohort of people with HIV receiving care at 14 clinic sites located in Washington, DC, and led by researchers at George Washington University (GW). The DC Cohort collects comprehensive data from the EHRs of consenting patients, which are subsequently aggregated into a centralized database on a monthly basis. At the study’s inception, a linkage to the DC Department of Health (DC Health) HIV surveillance data was proposed, with the goals of increasing the completeness and accuracy of both databases and identifying people enrolled at multiple DC Cohort sites [3]. Linked data include overlapping clinic and surveillance data, such as demographics, mode of HIV transmission, dates of diagnosis for HIV and AIDS, and HIV-related laboratory results, including CD4 T-lymphocyte (CD4) counts and HIV viral loads. Prior linkages relied on a series of SAS programs and were able to successfully increase the measurement of the proportion of persons retained in HIV care, increase the completeness of the data captured in the DC Cohort, and enumerate the number of sites where DC Cohort participants were receiving their HIV care [4].

While the linkage is a unique aspect of the DC Cohort, the process of linking data using complex SAS coding took an estimated 6 months to complete, thereby limiting the timeliness and utility of the linked data. To increase the ease, feasibility, accuracy, and timeliness of future linkages, the DC Cohort sought to identify a more efficient approach to data linkage and analysis. The ATra Black Box (Black Box henceforth) is a high privacy assurance technology used to match private health information across entities [5]. In 2015, Georgetown University (GU), funded by the National Institutes of Health (NIH), developed the Black Box to match HIV surveillance data across 3 US public health jurisdictions (Maryland, District of Columbia, and Virginia) with high rates of cross-migration for care. This initial pilot identified 21,472 potential duplicates from 161,343 HIV surveillance case records across the 3 jurisdictions [6]. These matches were validated by each health department using different methods, which found that over 95% of matches in the “exact” and “high” categories were true matches. A subsequent pilot project, funded by the US Centers for Disease Control and Prevention (CDC), expanded to an additional 6 jurisdictions and matched 290,482 cases from 799,326 uploaded records [7]. This project also included additional data fields that provided information on laboratory results and other key indicators needed by jurisdictions for public health action. In 2018, GU was funded by the CDC under PS18-1805 (Secure Data Sharing Tool to Support De-duplication of Cases in the National HIV Surveillance System) to work with 59 public health jurisdictions funded for HIV surveillance and prevention [8]. As of 2024, GU had enrolled 40 jurisdictions into this project, with quarterly runs of the Black Box to deduplicate cases and provide cross-jurisdictional data.

Based on the security, automations, and efficiencies of the Black Box, the DC Cohort partnered with the Black Box team to conduct DC Cohort-DC Health data linkages and to automate the processes for comparing data from DC Health and the DC Cohort. GU implemented updates to the Black Box architecture for this project, which allowed for additional flexibility with data uploads and analytics. This paper reports on the process and results of the initial linkage of the DC Cohort data with DC Health data in 2023, using this novel Black Box approach.

## Process of the Initial Linkage of Data

### Data Sharing Agreement

GU developed a data sharing agreement (DSA) to be signed by GW, GU, and DC Health. The DSA included language on the roles and responsibilities for each entity and included an appendix with a list of the variables to be shared and an appendix of the data security and confidentiality (DSC) guidelines for the project. The DSC document was based on the CDC National Center for HIV/AIDS, Viral Hepatitis, STD, and TB Prevention published standards for the sharing and protection of public health data [9]. The DSA was signed by all entities in March 2023. This study was reviewed and approved by the Institutional Review Boards at GW and DC Health. The Black Box project was found to be exempt from human subjects review by the GU Institutional Review Board.

### Security and New Architecture for the Black Box

The Black Box has a physically protected server, as it is located in a custom-designed rack at the Equinix data center in Virginia. Once locked, the system cannot be accessed by anyone from GU, except for the administrators in very specific instances, as described below. The server has no external connections to any device other than a power source. It saves data in temporary memory for data matching and is programmed with manual and automatic mechanisms for cleaning out memory in the event of unauthorized access. The Black Box is available only to authorized users through designated encrypted links using public/private keypair technology. Each user connecting to the Black Box has a unique public/private digital keypair for their designated compartment. The public key is sent to GU, while the private key resides on the user’s computer. The Black Box requires the public key to be preinstalled on the Black Box and validated by the corresponding private key for access. Encryption techniques are in compliance with the Advanced Encryption Standards, as outlined by the National Institute of Standards and Technology [10], to protect the highly sensitive data during transit between the jurisdiction and the Black Box System.

Prior to the DC Cohort project, the Black Box runs were completed using an architecture where the Black Box programming was hard-coded for each run, and changes to the data or outputs required expertise in the Ada programming language [11]. This limited the type of analytics that could be programmed and did not allow for quick changes to data file inputs, as data systems change over time. Updates to the Black Box programming could only occur when the Black Box was unlocked. Locking down the Black Box required representatives from the entities sending private data to physically travel to the secured facility where the Black Box is housed. In the presence of GU staff, the Black Box was secured with keys, and the management cable was disconnected, which provided access for GU IT staff. The Black Box had to be unlocked after each run to allow access from GU to the machine after the data were erased.

In 2021, GU updated the system to include a number of new features that allowed the Black Box programming to be more generic and easily adapted to different types of projects. These updates included implementing a templating system that would allow a Black Box administrator at GU to create project-specific files. These files, uploaded to the Black Box, specify the expected inputs, the matching algorithm, and the reports the Black Box will generate. These templates can be quickly changed by the administrator without having to unlock the Black Box. GU also added functionality to how data specific to the DC Cohort project are shared in the Black Box. For example, the PROXY function allowed the sharing of data without any protected health information to the DC Cohort study team. This was a significant change from previous iterations of the Black Box, which shared data between public health jurisdictions that are authorized to view protected health information.

GU also implemented a new user interface (Black Box client) to make it easier for the end user to connect to the Black Box and allow GU to add analytic plug-ins to create tailored output reports for users. For this project, GU used a plug-in for SAS software [12]. GU developed SAS codes for the output for both the DC Cohort and DC Health. These codes included comparisons of key demographic variables and determining which laboratory results were new to each entity while formatting variables for easier input into the DC Cohort and DC Health systems.

### Data Collection

The HIV surveillance system used by most US public health jurisdictions, the enhanced HIV/AIDS Reporting System (eHARS), is a document-based surveillance system that allows all documents to be stored and retained electronically in their original format. The document view in the eHARS contains all documents for each person. The person view provides a summary record for each person, derived from all entered records for that person, and uses a hierarchy to determine data elements with multiple entries in the document view. Each record in the document view may contain variations of a person’s identity, including misspellings of the person’s first, middle, and last name; a reverse name order; typographical errors in the date of birth (DOB); and potential aliases. GU validated matches using all variations of first and last names (ie, alias names) recorded in the eHARS in another project [13] and added these name variations to the algorithm for matching for the DC Cohort. Other data from the eHARS and DC Cohort for this project include demographics, opportunistic infection history, dates of HIV and AIDS diagnoses, CD4 counts and percentages, and viral loads.

### Matching Algorithm

The final algorithm used for the linkage to match records in the Black Box (Table 1) was based on those used in other Black Box projects and the previous DC Cohort linkages [4,6]. Variables used for matching included first name, last name, DOB, Social Security number (SSN), and birth sex. Match-level scores ranged from 20 (ie, a match on Cohort ID only) to 100 (ie, a match on first name, last name, DOB, SSN, and birth sex). The matching algorithm was reviewed following the validation results of the initial test runs. Changes included removing match levels with few or no matches and increasing the score for match levels with a high percentage of valid matches. A threshold score of 61 was established after the validation, allowing DC Health data with that score level and higher to be shared with the DC Cohort. More details on the validation process are provided below.

**Table 1.** DC Cohort ATra Black Box matching algorithm (July 2024).

Name variables	Other match variables	Score
First name and last name (both person view and alias names in DC Health^a^ data)	Cohort ID^b^, DOB^c^, SSN^d^, and birth sex	100, 99^e^
First name and last name (both person view and alias names in DC Health data)	DOB, SSN, and birth sex	98, 97^e^
First name and last name (both person view and alias names in DC Health data)	Cohort ID, DOB, partial SSN^f^, and birth sex	95, 94^e^
First name and last name (both person view and alias names in DC Health data)	DOB, partial SSN, and birth sex	93, 92^e^
First name and last name (both person view and alias names in DC Health data)	Cohort ID, DOB, and SSN	90, 89^e^
First name and last name (both person view and alias names in DC Health data)	DOB and SSN	88, 87^e^
First name and last name (both person view and alias names in DC Health data)	Cohort ID, DOB, and partial SSN	86, 85^e^
First name and last name (both person view and alias names in DC Health data)	DOB and partial SSN	84, 83^e^
First name and last name (both person view and alias names in DC Health data)	Cohort ID, DOB, and birth sex	82, 81^e^
First name and last name (both person view and alias names in DC Health data)	DOB and birth sex	80, 79^e^
First name and last name (both person view and alias names in DC Health data)	Cohort ID and DOB	78, 77^e^
First name and last name (both person view and alias names in DC Health data)	Cohort ID and SSN	76, 75^e^
First name and last name (both person view and alias names in DC Health data)	DOB	74, 73^e^
First name and last name (both person view and alias names in DC Health data)	SSN	72, 71^e^
First name and last name (both person view and alias names in DC Health data)	Cohort ID and partial SSN	70, 69^e^
First name and last name (both person view and alias names in DC Health data)	Partial SSN	68, 67^e^
Last name (person view only)	Cohort ID, DOB, and SSN	66
Last name (person view only)	DOB and SSN	65
Last name and first name (first 6 letters; person view only)	Cohort ID and DOB	64
Last name and first name (first 6 letters; person view only)	DOB	63
None	Cohort ID, DOB, and SSN	62
None	DOB and SSN	61
Last name (person view only)	DOB	59
Last name (person view only)	Cohort ID	58
None	Cohort ID, DOB, and partial SSN	40
None	DOB and partial SSN	38
None	Cohort ID and DOB	30
None	Cohort ID	20

a DC Health: DC Department of Health.

b Cohort ID: participant study ID.

c DOB: date of birth.

d SSN: Social Security number.

e The higher score is for a match to the person view name, and the lower score is for a match to an alias name.

f Partial SSN: last 4 digits of the Social Security number.

### Initial Linkage

**Figure 1.**
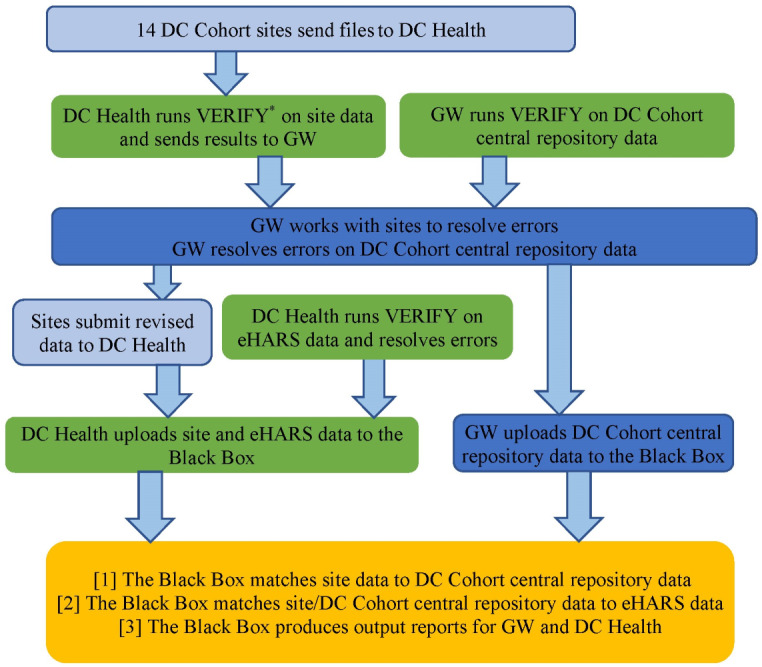
Process of Black Box linkage for the DC Cohort. Black Box: ATra Black Box; DC Health: DC Department of Health; eHARS: enhanced HIV/AIDS Reporting System; GW: George Washington University. *VERIFY is a software program developed by Georgetown University to check files before they are uploaded to the Black Box. It checks each data file against an internal template and notifies the user of any errors in the data files.

In the initial linkage of the GW and DC Health data using the Black Box, the 14 DC Cohort sites sent files using a secure file transfer protocol to DC Health, which contained the matching information (name, DOB, and SSN) for all persons enrolled in the study through a specific date (Figure 1). For this linkage, the end date was September 30, 2022. A DC Health staff member reviewed the site files and worked with GW and the sites to resolve any issues. DC Health staff uploaded data from the sites and DC Health to the Black Box, while a GW staff member uploaded data on study participants from the DC Cohort central repository. The central DC Cohort data represent a limited dataset that does not contain all identifiers for a person and only includes basic demographic information (eg, state of residence, sex at birth, and race), testing information (eg, date of visit, test type, and test result), and the Cohort ID to uniquely identify the records. The Black Box first ran a check on the Cohort IDs in the DC Cohort data against those in the site files. Only records for persons with data in both the DC Cohort data and site files were retained by the Black Box for matching with the DC Health data. The records that did not match between the DC Cohort data and site files were sent to GW and DC Health by the Black Box in 2 filter reports: one that provided the Cohort IDs that were in the DC Cohort data but not the site files and one that provided the Cohort IDs that were in the site files but not the DC Cohort data. The matching algorithm was run on the filtered site data and DC Health data. The Black Box produced a number of reports that are outlined below. The first run occurred on April 28, 2023.

After the initial runs of the Black Box, the staff at DC Health performed data validation of the match results in late 2023, using Link Plus 2.0, a standalone probabilistic record linkage software created by the CDC [14]. Link Plus pairs records from each file, calculates a score indicating the likelihood that each record refers to the same person, ranks the matched pair based on the score, and highlights discrepancies in each record to help identify true matches and discard incorrect ones. The last run for the initial linkage occurred on July 1, 2024, and took 14 minutes for the Black Box to complete.

### Reports From the Black Box

The Black Box client produces reports designed by GU that are specific for the DC Cohort and DC Health (Table 2). For the DC Cohort, the reports only contain the Cohort ID. DC Health receives a full match report with the matching identifiers, including name, DOB, SSN, etc. The Black Box also generates an unmatched report, which lists all the Cohort IDs for the DC Cohort that did not match to DC Health data and the reasons (ie, they were not in the site data or they did not match to the eHARS data).

**Table 2.** DC Cohort ATra Black Box reports (July 2024).

Report	Recipients	Description
Filter reports	DC Cohort and DC Health^a^	Two reports: (1) Cohort IDs^b^ from the DC Cohort central file that did not have matching Cohort IDs in the site files, and (2) Cohort IDs from the site files that did not have matching Cohort IDs in the DC Cohort central file. These Cohort IDs are excluded from the match with DC Health.
Match reports	DC Cohort and DC Health	Cohort IDs from the merged DC Cohort/site file that matched to DC Health at any score level. The report for DC Health includes the DC Health unique identifier, the matching score, and all the matching variables. The report for the DC Cohort includes only the Cohort ID and the matching score.
Unmatched reports	DC Cohort and DC Health	Cohort IDs from the merged DC Cohort/site file that did not match to DC Health at any score level.
DC Cohort multiple site report	DC Cohort and DC Health	Cohort IDs from the merged DC Cohort/site file that matched to DC Health, where multiple Cohort IDs matched to the same unique identifier at DC Health.
DC Cohort unmatched report	DC Cohort	Cohort IDs from the DC Cohort file that were either (1) filtered out because they did not have a matching Cohort ID in any of the site files or (2) were not filtered out but did not match to DC Health.
DC Cohort lab site report	DC Cohort	Facilities for lab records that matched to a Cohort ID. These data are used to determine if a person is receiving care at a Cohort site.
Demographics report	DC Cohort and DC Health	Demographic data for all persons who matched between the merged DC Cohort/site file and DC Health at the threshold score or above. There is a record for every matched Cohort ID, and demographic data are populated if the data are missing or discrepant between DC Health and the DC Cohort or if the variable does not exist in one of the files. For example, if the HIV diagnosis date is different in the 2 files, both the DC Cohort and DC Health will receive the value for the other entity.
DC Health opportunistic infections for the DC Cohort	DC Cohort	DC Health data for opportunistic infections for all persons who matched between the merged DC Cohort/site file and DC Health. There is a record for every matched Cohort ID that has opportunistic infection data at DC Health.
Laboratory results	DC Cohort and DC Health	Lab records for CD4^c^ count, CD4 percentage, and viral load for records that match above the score threshold. Lab records are compared by Cohort ID, sample date, lab type, result, and result interpretation, and then coded into a lab group based on how many variables matched. Lab records must occur after the DC Cohort consent date.
Treatment report	DC Health	Data file that provides antiretroviral treatment information from the DC Cohort data for all persons who matched between the merged DC Cohort/site file and DC Health above the score threshold.

a DC Health: DC Department of Health.

b Cohort ID: participant study ID.

c CD4: CD4 T-lymphocyte.

The DC Cohort team receives a report on study participants who matched to the same case record in the eHARS, indicating that these Cohort IDs represented the same person who may have enrolled in the DC Cohort study at more than one DC Cohort site. The demographics report uses SAS code to provide data for matched cases at scores of 61 and above. This report compares demographics between DC Health and the DC Cohort and reports on any discrepancies found for variables, including vital status, gender, sex at birth, race, HIV transmission risk, and current state of residence. The DC Cohort team receives a report on all of the opportunistic infections that were found in the eHARS for matched participants.

Additionally, a lab report provides CD4 counts, percentages, and viral loads that were not found in both sets of data when compared by combinations of lab date, lab type, result, and result interpretation. After the initial runs of the Black Box, it was determined that there were potential differences in how lab data were recorded in each system, and a lab grouping variable was created to indicate which variables matched and did not match between the 2 systems.

## Findings of the Initial Linkage of Data

**Figure 2.**
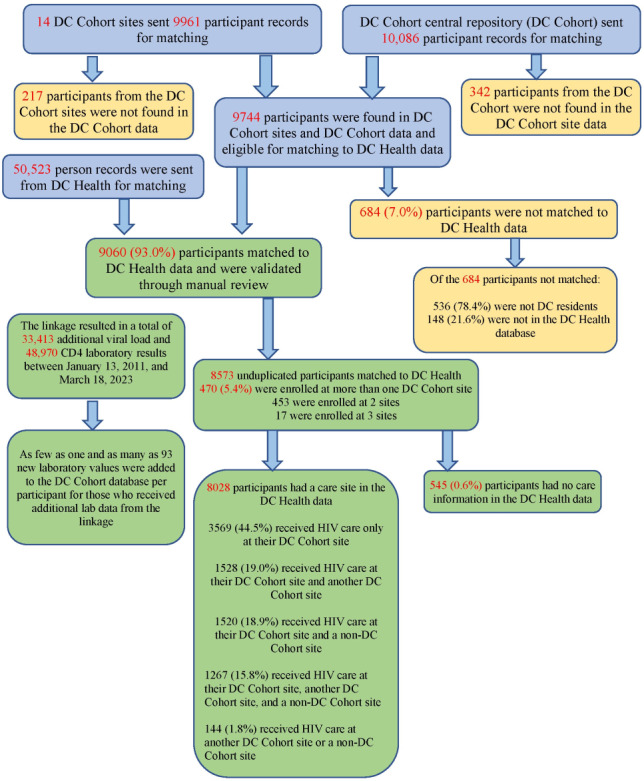
Summary of the results of ATra Black Box linkage of the DC Cohort. DC Health: DC Department of Health.

In the initial linkage, a total of 9961 records were uploaded from the 14 sites and 10,086 records from the GW DC Cohort database in July 2024 (Figure 2). Of these, 342 records were in the GW data but not in the site files and 217 records were in the site files but not in the GW data, resulting in 9744 study records eligible to be matched to the DC Health data. The majority of the records that did not exist in both datasets were related to records for site participants who were enrolled after the consent cutoff date (September 30, 2022) and records that came from a specific DC Cohort site.

During the validation process, uncertain matches were manually reviewed, and pairs were confirmed as true matches when there was no ambiguity that both records belonged to the same person. The DC Health staff completed a full validation of the matches in the initial linkage and found that 98.7% (9257/9378) of matches were true matches. Some matches were deemed false matches, and a few remained uncertain. If uncertainty could not be resolved, even after cross-checking records, the pair was flagged as a false match. DC Health used other available databases to check the matches, which included the Chesapeake Regional Information System for our Patients, a regional health information exchange, and the DC Public Health Information System that houses a number of public health surveillance datasets for DC. The majority of false matches (105/121, 86.8%) were found at scores below 60. Based on this manual data validation using Link Plus, the matching algorithm and threshold for sharing data were revised.

Of the 9744 records eligible for matching, 9060 (93.0%) matched at a score of 61 or above and were eligible for sharing data between the DC Cohort and DC Health databases. Of the 684 records that did not match, 536 (78.4%) were not DC residents and the remaining 148 (21.6%) were not found in the DC Health database. After deduplicating persons across DC Cohort sites, 8573 unique persons matched between the DC Cohort and DC Health.

The Black Box linkage added 33,413 viral load lab records and 48,970 CD4 count records to the DC Cohort database, with a range of 1 to 93 lab records added per DC Cohort participant. This was an improvement from the previous linkage that found 10,252 additional CD4 count records and 5948 additional viral load records [4].

The linkage also provided information on the sites where DC Cohort participants received HIV care and identified 8028 (93.6%) participants with a care site in the DC Health data. Among participants for whom a clinic site was identified, 44.5% (3569/8028) received care only at the DC Cohort site where they enrolled in the study, 53.7% (4315/8028) received care at multiple sites, and 1.8% (144/8028) received care at a different DC Cohort site or a non-Cohort site.

The DC Health data also provided additional information on deaths for persons in the DC Cohort. The initial Black Box linkage added death dates for 767 persons in the DC Cohort who were not previously identified as deceased. As part of routine HIV surveillance, DC Health conducts annual matching with the National Death Index and the Social Security Death Master File and matches data with local vital statistics databases. Cause of death information from the eHARS was also shared for DC Cohort participants. Death dates and cause of death information were incorporated into the DC Cohort data system, and death dates were visible to the DC Cohort site personnel. The previous linkage found an additional 57 deaths for DC Cohort participants, and thus, the use of the Black Box greatly improved the accuracy of vital status information for the DC Cohort [4].

The linkage also identified participants who were seen at multiple DC Cohort sites. Because GW only receives the Cohort ID and does not receive the participant’s name or other identifying information, GW staff are not able to determine if a person is enrolled at multiple sites. Therefore, to identify participants receiving care at multiple sites and deduplicate records, the Black Box reports provided deidentified information by listing multiple DC Cohort IDs that matched to the same person at DC Health. The initial run found that 470 (5.4%) of the 8573 unduplicated participants were enrolled at more than one DC Cohort site. Of these 470 persons, 453 were enrolled at 2 DC Cohort sites and 17 were enrolled at 3 DC Cohort sites.

## Discussion

### Summary

We were able to successfully modify a novel privacy technology, the Black Box, to facilitate the linkage of EHR-based study data and HIV surveillance data in Washington, DC. The matching process involved several refinements and was eventually able to provide useful information to more accurately describe DC Cohort participants and key HIV data, including the capture of HIV-related lab records, vital status, and care patterns.

While the initial design and refinement of the Black Box algorithm and reporting required extensive time to implement, the actual matching process time was greatly reduced from previous linkages. Completing the algorithm, executing the DSA, running multiple tests, fixing coding issues, manually reviewing thousands of records to verify the Black Box matches, and revising the scoring system took approximately 18 months. Staff members at DC Health and GW were required to install the Black Box client software and received technical onboarding from the GU staff on uploading the data and retrieving the reports. However, once the data were uploaded, the matching process took less than 15 minutes, and reports were available immediately. The implementation of the SAS plugin scripts aided in data comparisons and reduced the amount of postanalytic code needed, although some data quality and verification checks were still needed.

The high percentage of persons found in both the DC Cohort and DC Health datasets (9060/9744, 93.0%) indicates a robust matching algorithm that will reduce the need for manual review in future linkages. The prior linkage method identified 91% of persons who were in both datasets. The implementation of the Black Box greatly improved the data linkage process compared with manual methods used in the past. Past linkages required extensive manpower to run the matching and conduct a manual review to ensure accuracy. The automation provided by the Black Box has reduced this burden, streamlining the process, minimizing human error, and increasing efficiency. The Black Box linkage found a higher percentage of deaths reported in the DC Health data than previous linkages and provided a much higher number of CD4 counts and viral loads than previous linkages.

### Limitations

The deterministic algorithms used by the Black Box have some limitations. A recent study found that probabilistic algorithms detected more matches than the Black Box matching algorithm used in the NIH pilot when matching surveillance data for HIV and sexually transmitted infections [15]. For this project, an important factor in matching is ensuring that health data are correctly associating a person, because these data are used for many purposes, including locating people out of care. This linkage examined false match rates and found them to be less than 0.1% for matches above a score of 61. DC Health also compared linkage results from the Black Box with the results from a probabilistic matching software, Link Plus, and found over 99% congruence at match levels above 60.

### Conclusions

This project was able to match data between a research cohort and a public health surveillance dataset with a high degree of accuracy and provide key data elements to characterize the cohort and the health outcomes over time. The implementation of the Black Box for sharing HIV surveillance and DC Cohort data resulted in improved data quality and decreased the time needed by staff to resolve conflicts. The approach allows the DC Cohort and DC Health to update data on care status and other key indicators by using data from the Black Box. The Black Box data provide a more comprehensive picture of care delivery for people with HIV compared with clinical records alone and allow tracking of care patterns over time. It has improved the timeliness, accuracy, and completeness of key HIV data that are needed to measure progress on HIV and “Ending the HIV Epidemic in the US” indicators at the local and national levels. The use of these combined data will improve accuracy regarding HIV surveillance for the health department, improve measurement of the care continuum and clinical outcomes, allow us to answer additional research questions about care patterns and co-morbidities, and help develop evidence-based interventions to improve outcomes among people with HIV.

Future linkage plans include adding data elements to the exchange, including co-morbidities such as sexually transmitted infections, hepatitis, and tuberculosis. Linkage with other datasets, including DC Medicaid and Cancer Registries, is also planned.

## ABBREVIATIONS

CD4: CD4 T-lymphocyte
CDC: US Centers for Disease Control and Prevention
DC Health: DC Department of Health
DOB: date of birth
DSA: data sharing agreement
DSC: data security and confidentiality
eHARS: enhanced HIV/AIDS Reporting System
EHR: electronic health record
GU: Georgetown University
GW: George Washington University
NIH: National Institutes of Health
SSN: Social Security number
